# 

**Published:** 1901-04-20

**Authors:** 


### NOTICE TO CORRESPONDENTS.

All MSS., Letters, Books for Review, and other matters intended for the Editor should be addressed The Editor, "The Hospital," The Hospital Building, 28 & 29 Southampton Street, Strand, W.C.

The Editor cannot undertake to return rejected MS., even when accompanied by stamped directed envelope.

**Notice to Advertisers and Subscribers.**

All Advertisements, Orders for Copies of The Hospital, and Business Communications should be addressed to **THE MANAGER (Not to the Editor),** The Hospital, 28 & 29 Southampton Street, Strand, London, W.C.

The Cost of Subscription, post free, is as follows:—Per week, 2½d.; per quarter, 2s. 8d.; per half-year, 5s. 3d.; per annum, 10s. 6d. Foreign:—Per annum, 15s. 2d., payable, in all cases, in advance.

**NOTICE.—Vol. XXIX. of "The Hospital" (October 1900, to March, 1901), in handsome cloth binding will shortly be on sale at the Office of this Journal, price, 7s. 6d. Cases for binding the Numbers contained in this Volume 1s. 6d. Index to Vol. XXIX., 2½d., post free.**

**The Monthly Part for April is now ready. Price 6d., by post 8d.**

### BOOKS RECEIVED.

Hodder and Stoughton.

"The Self-educator in Chemistry." By James Knight, M.A., B.Sc., F.C.S. Edited by John Adams, M.A., B.Sc.

H. K. Lewis.

"Hygiene and Public Health." By Louis Parkes, M.D., D.P.H.Lond. Univ., and Henry Kenwood, M.B., D.P.H., F.C.S.

"A Contribution to the Study of the Blood and Blood Pressure." By George Oliver, M.D.Lond., F.R.C.P.

Cassell and Co.

"Difficult Labour." By G. Ernest Herman, M.B.Lond., F.R.C.P. New and revised edition.

R. Morgan.

"The British Weather Chart." By B. G. Jenkins, F.R.A.S.

Albert Broadbent.

"Fruits, Nuts and Vegetables. Science in the Daily Meal." By Albert Broadbent.

### SCRAPS AND GLEANINGS.

His Majesty the King has consented to continue his Patronage to the Hospital for Consumption and Diseases of the Chest, Brompton.

The estimated cost of the proposed extension of the Nurses' Home at the Hull Royal Infirmary, including furnishing, is £4,000.

In 1900 there were 532 in-patients in the Tunbridge Wells General Hospital, as against 559 in 1899. The out-patients in 1900 numbered 4,465.

The Ladies' Collecting Committee collected a sum of £162 during the year 1900 for the benefit of the Hastings and East Sussex Hospital.

During the past year 4,335 patients were treated at the Manchester Southern Hospital, the total expenditure was £3,374, the income being £2,419.

The Duchess of Portland is heading a movement on the part of the City and County of Nottingham to endow a cot in the Nottingham Children's Hospital in memory of Queen Victoria. A sum of £1,000 is required to endow the cot.

During the past year the receipts of the Cirencester Cottage Hospital were £572, and the expenditure £424, leaving a balance in hand of £148. The donations showed an increase of £50. The Hospital Sunday collections amounted to £136 odd.

At a recent public meeting at Bournemouth, convened in support of the proposed memorial to Queen Victoria, a resolution to adequately endow the beds in the Empress Victoria Ward of the Royal Boscombe and West Hants Hospital was unanimously adopted.

The report of the Women's and Children's Hospitals, Redruth, shows that the number of patients during the year had been 142, of whom 110 were women and 32 children. The average cost per head including the staff was £29, the average cost per head for food alone being £13.

The Hospital Sunday collections at Southampton during the ten years ended 1900 amounted to upwards of £11,000. The collection in 1900 amounted to £1,165, of which the churches and chapels contributed £706; the employees of twenty-two different firms in the town, £88; the Union Castle Steamship Co.'s ships, £160; and the school children, £21.

The report of the Hull Royal Infirmary for 1900 shows that 17,212 patients were treated during the year, as against 18,994 the previous year, there being an increase of 41 in-patients and a decrease of 1,823 out-patients. The receipts for income in 1900 show an increase over any one of the preceding five years, but the expenditure for the year exceeded the income by £319.

A Masonic Lodge has been founded in connection with the Middlesex Hospital, and the consecration will take place on Tuesday, May 7th. T. G. A. Burns, Esq., M.A., P.M., P.P.G.D. (Surrey) has been elected Worshipful Master, and has appointed W. Duncan, Esq., M.D., Senior Warden; and F. J. Wethered, Esq., M.D., Junior Warden. Henry Morris, Esq., F.R.C.S., will act as treasurer, and F. Clare Melhado, Esq., secretary.

### THE STUDENT OF MEDICINE.

### APPOINTMENTS.

**H. C. Allinson**, M.R.C.S.Eng., appointed Medical Officer of Health for the Borough of King's Lynn. **George Ashton**, M.B., Ch.B.Vict., M.R.C.S.Eng., L.R.C.P.Lond., appointed Senior House Surgeon to the Bolton Infirmary. **R. Hamilton Bell**, M.A., M.B., B.C.Cantab., appointed Physician to Out-patients, Samaritan Free Hospital for Women and Children. **F. W. Beville**, M.R.C.S.Eng., L.R.C.P.Lond., appointed Medical Officer to the Uxbridge Union Workhouse. **Thomas**

**Robert Bradshaw,** B.A., M.D.Univ.Dub., M.R.C.P.Lond., appointed Physician to the Liverpool Royal Infirmary. **Harry Haward Bywater,** M.B., Ch.B.Vict., appointed Senior House Surgeon to the Preston Royal Infirmary. **A. Charpentier,** M.D.Durh., appointed Medical Officer of Health for the Uxbridge Rural District. **G. Clingan,** M.D.Toronto, appointed Clinical Assistant to the Chelsea Hospital for Women. **J. H. Crocker,** M.D.Vict., D.P.H., appointed Medical Officer of Health for the Borough of Richmond, Surrey. **R. G. Cross,** L.R.C.P.Lond., M.R.C.S.Eng., D.P.H., appointed Medical Officer of Health for the Petersfield Rural District, and District and Workhouse Medical Officer of the Petersfield Union. **W. Edmunds,** M.A., M.C.Cantab., F.R.C.S., appointed Honorary Surgeon to the Tottenham Hospital. **G. W. Eglinton,** L.R.C.P.Edin., &c., appointed Medical Officer of Health to the Wells Rural District Council. **Hugh Galt,** M.B., C.M., F.F.P.S.G., D.P.H.Camb., appointed Medico-Legal Examiner for the Crown in criminal cases occurring in Glasgow and the Lower Ward of Lanarkshire. **Wilfrid Glegg,** M.D., M.R.C.P.Edin., appointed Senior Resident Medical Officer to the Throat Hospital, Golden Square, London, W. **O. F. F. Grunbaum,** M.A., B.C.Cantab., D.Sc.Lond., appointed Clinical Pathologist to King's College Hospital. **Walter M. Hamilton,** M.D., D.P.H., appointed Medical Officer of Health for the Borough of Eccles. **Philip Edward Hill,** M.R.C.S.Eng., L.S.A.Lond., appointed Medical Officer of Health for the Crickhowell Combined Districts. **G. F. Hulme,** M.B., C.M., appointed Medical Officer of Health for the Urban District of Felixstowe and Walton. **W. A. S. Lamborn,** M.R.C.S.Eng., L.R.C.P.Lond., appointed Assistant to the Medical Superintendent of the Highgate Hill Infirmary of the Parish of St. Mary, Islington. **W. K. Loveless,** L.R.C.P., L.R.C.S.Edin., appointed Medical Officer of Health for the Stockbridge Rural District. **Philip Macdonald,** M.B., Ch.B.Edin., appointed Junior House Surgeon to the Preston Royal Infirmary. **Basil Middleton,** M.B., Ch.B.Vict., appointed Assistant House Surgeon to the Preston Royal Infirmary. **W. E. Nelson,** M.R.C.S.Eng., L.R.C.P.Lond., appointed Medical Officer for the Wootton Waven District of the Stratford-on-Avon Union. **H. Reginald Pring,** M.R.C.S., L.R.C.P., L.D.S.Eng., appointed Demonstrator to the Dental Hospital of London and London School of Dental Surgery. **Alan Butler Slater,** M.B., Ch.B.Edin., M.R.C.S., L.R.C.P.Lond., appointed House Surgeon to the Hospital for Women and Children, Leeds. **Lewis A. Smith,** M.B.Lond., M.R.C.P., appointed Physician to Poplar Hospital. **Miss Lucy E. Smith,** M.B., B.Ch., B.A.O., R.U.I., appointed Junior Assistant Physician to the Erinville Lying-in Hospital, Cork. **E. Taunton,** M.R.C.S.Eng., L.R.C.P.Lond., appointed Senior Assistant Medical Officer of the St. Pancras Workhouse, Pancras Road. **T. Telford Smith,** M.D.Dub., appointed District Medical Officer of the Wimborne and Cranborne Union. **W. H. Walter,** M.D.Brux., L.R.C.P., M.R.C.S.Eng., L.S.A.Lond., appointed Medical Officer to the Brentford Cottage Hospital and Dispensary. **W. J. Watkins,** L.S.A., appointed Medical Officer of Health for the Bolsover Urban District. **A. J. Whiting,** M.D.Edin., M.R.C.P.Lond., appointed Honorary Assistant Physician to the Tottenham Hospital.

